# Evaluation of geriatric head and neck trauma cases in a German highest-level trauma centre from 2018 to 2024

**DOI:** 10.1186/s12877-025-06790-z

**Published:** 2025-11-25

**Authors:** Ákos Bicsák, Jens-Peter Stahl, Leonie Koch, Evangelos Vitkos, Stefan Hassfeld, Lars Bonitz

**Affiliations:** 1 Department of Health, University Witten/Herdecke, Alfred-Herrhausen-Straße 50, Witten, D-58455 Germany; 2 Department of Cranio- Maxillofacial Surgery, Regional Plastic Surgery, Dortmund General Hospital, Chair of the University Witten/Herdecke, Muensterstrasse 240, Dortmund, 44145 Germany; 3 Department of Traumatology, Dortmund General Hospital, Muensterstrasse 240, Dortmund, D-44145 Germany

**Keywords:** Head and neck injury, Facial bone fracture, Elderly, Geriatrics, Mandible, Maxilla

## Abstract

**Background:**

Geriatric populations face heightened vulnerability due to aging, socioeconomic challenges, and marginalization. In 2023, 18.1% of Germans over 65 were at risk of poverty, with women and those aged 80 + disproportionately affected. The SARS-CoV-2 pandemic (2020–2022) exacerbated these issues, restricting healthcare access. This study investigates geriatric head and neck trauma trends (2018–2024) across pre-pandemic, pandemic, and post-pandemic periods.

**Methods:**

Conducted at a level 1 trauma centre, data were sourced from the Dortmund Maxillofacial Trauma Registry (14,500 + cases, 2007–2024). Fractures were categorized per AO guidelines, and demographic analyses, fracture distribution and other medical conditions were presented.

**Results:**

This study analysed 973 geriatric head and neck trauma cases (2018–2024). Falls were predominant (82.6%), with significant comorbidities (2.6 per patient), including hypertension (64%). Males experienced a 73% decrease in case numbers in 2021. Injuries primarily affected the midface, with 52% requiring surgery. Pandemic disruptions influenced trends, highlighting geriatric trauma complexity and care demands are detailed analysed.

**Discussion:**

Our study presents comprehensive data on geriatric head and neck traumatology, analyzing 65–75 y.o. and > 75 y.o. groups. Male predominance was significant at 65–70 y.o. (*p* < 0.001) but reversed above 75 y.o., with significant female dominance. Since the SARS-CoV-2 pandemic, case numbers showed extreme volatility, with a 30% rise in females and 90% in males in 2021, followed by a record low in 2022. Cerebral injuries occurred in 49.3% of patients, with 2.6 comorbidities per capita and 52% requiring surgery. Anticoagulant therapy was frequent (≈ 74% antiplatelet, 26% anticoagulant). Falls caused 82% of injuries, predominantly affecting the nasal bone, zygomatic bone (218), and orbital floor (262), indicating high vulnerability in this population.

**Conclusion:**

Our study reveals volatility in case numbers post-SARS-CoV-2, with males disproportionately affected. A high proportion of elderly patients take anticoagulants. Among the injured, there is a high rate with cerebral injury. Most fractures occur in exposed facial regions. Ongoing surveillance is essential to address increasing geriatric vulnerability.

## Background

The geriatric population is considered one of the most vulnerable populations [1–3]. This population is affected by aging and socioeconomic status, an increasing proportion of women, and social marginalization. As per current data of 2023, 18.1% of Germans above the age of 65 are exposed at risk of poverty [4]. The situation is even worse among the population of 80 years and older: 22.4% are expected to face poverty. Females and people with low educational levels are more affected [4].

From 2020 to 2022 the SARS-CoV-2 pandemic led to extreme situations in Germany, too. There were many lockdowns affecting not just family life but also limiting access to healthcare facilities, medical care, and social system institutions [5–7]. In the city of Dortmund, more than 43.000 SARS-CoV-2 positive cases were recorded since the outbreak, with most cases were recorded from January 2021 to December 2022 [8].

As the head and neck region is particularly exposed to injuries, it is essential to reconsider the current trends and developments of the situation. Our study aimed to provide an update on the trends in geriatric head and neck traumatology from 2018 to 2024. We defined pre-pandemic, pandemic, and post-pandemic period with each 2 years of time, as accessibility to the healthcare system and its processes shows three clearly different characteristics.

## Methods

### Treatment procedures

Our hospital is the highest-level interregional trauma centre responsible for approximately 3 million people who may sustain head and neck injuries. All patients with head and neck injuries are admitted to the Emergency Department, led by the Department of Traumatology. The necessary body checks and initial physical examinations are performed by trained trauma surgeons. Emergency radiologist, neurosurgeon, oral- and maxillofacial surgeon, intensive care specialists, geriatricians, eye specialists and so an are available 24/7/365.

After the initial examination, patients are directed to the leading Department; treatment plans are interdisciplinary, created, and evaluated. The injuries are classified, and the treatment process is organized based on the most severe injuries. Each Department performs its standard treatment based on guidelines.

### Classification of fractures, databank, statistics

The Dortmund Maxillofacial Trauma Registry is a Redcap-based [9, 10] database that serves to analyse our patient data. A total of more than 14,500 patients were included from 2007 to 2024. The database was locked on the 30th of September, 2024.

The definition of the geriatric age group is not unified in international literature. Multiple, age related diseases, other conditions influence the classification of the individual patients into the geriatric group. Chronologically, the German Society of Geriatrics (DGG), the German Society of Gerontology and Geriatrics (DGGG), and the German Group of Geriatric Institutions (BAG) define the geriatric patient as 65 years or older. In our study, we used this classification [11].

The fracture classification follows the widely accepted AO Classification supplemented with the “fracture of the anterior wall of the maxillary sinus” [12]. The data regarding demographics, medical history, concomitant medication, fracture data, and data on conservative or operative treatment were collected for each case.

The study inclusion criteria comprised all elderly patients 65 years old or older, who sustained head and neck injuries and were listed in our Maxillofacial Trauma Registry. The further data processing was performed on this geriatric database. In each of these cases, the actual number containing data from January to September 2024 (9 months) was multiplied by $\:\left(\frac{12}{9}\right)$ (supposing an approximately similar distribution within the year) and marked with italics and an asterisk (*). The distribution of data was checked using Kolmogorov-Smirnov and Shapiro-Wilk tests. We perfomed descriptive statistics on the demographic data. Group comparisons with χ2-test for normally distributed data (*p* < 0.05 for significance) and Wilcoxon ranked test for non-normally distributed data (*p* < 0.05 threshold for significance) were used. The rounding of the counted values was performed as per mathematical standards. We used SPSS 29.0 (IBM^®^) for the statistical analysis. We presented the results in text form, tables, diagrams, and a heatmap.

## Results

The Ethics Commission of the University of Witten/Herdecke has approved this study (No. 152/2017). It was conducted in accordance with the Helsinki Declaration, the laws and regulations of the European Union, the Federal Republic of Germany, the State North-Rhine-Westphalia, and the General Hospital Dortmund.

The Dortmund Maxillofacial Trauma Registry database lock was performed on the 30th of September, 2024. In the period from the 1 st January 2018 to the database lock, there were 6971 primary injured patients and 544 secondary cases, taken together, this corresponds to a total of 7515 cases over seven years. Among the primary cases, 965 geriatric patients (13.8%) were identified. In some cases, 2024 data was rounded up to cover the full year, these are marked in Italics and with an asterisk (*).

**Fig. 1:**
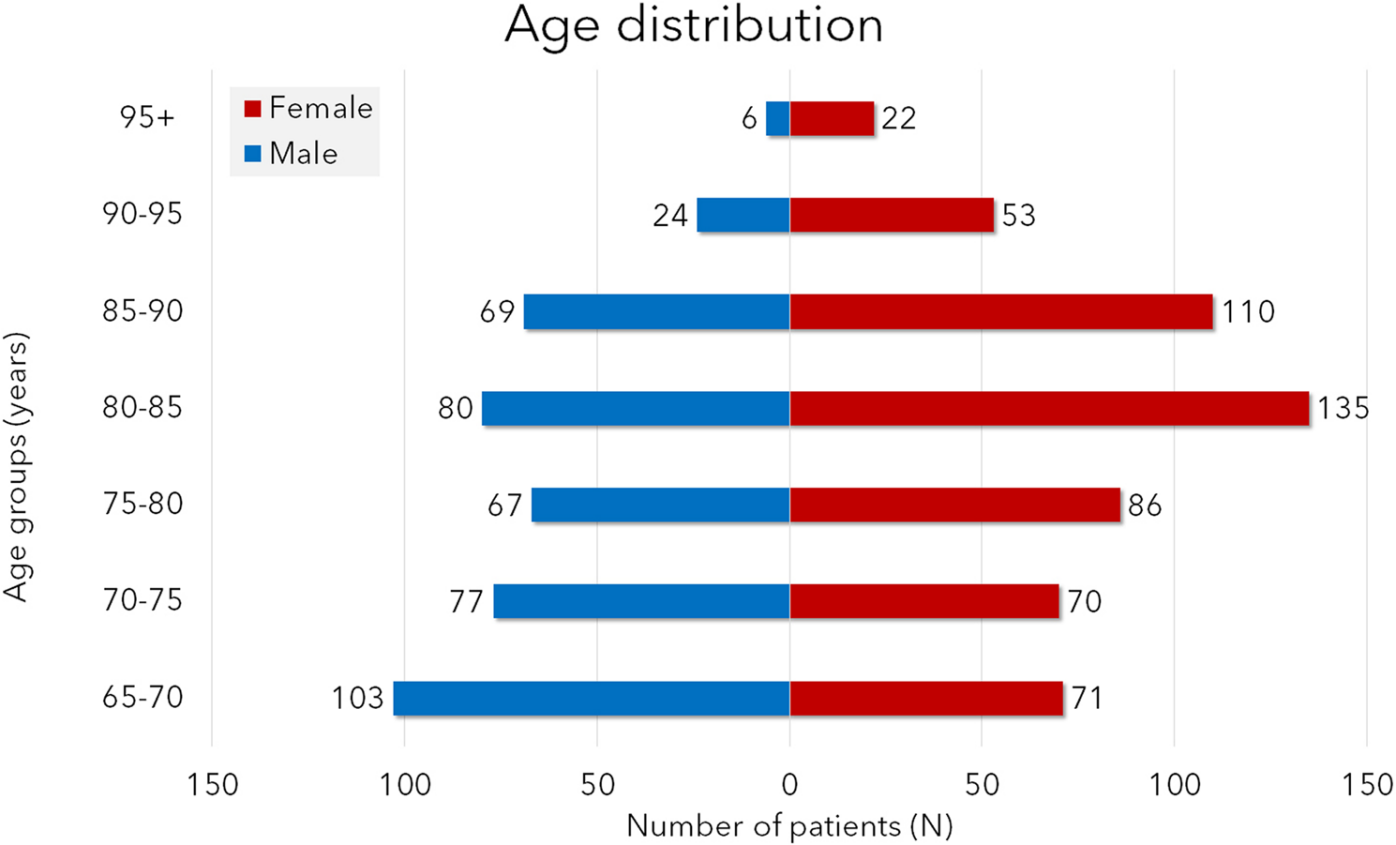
Histogram showing the age and sex distribution of geriatric patients (N, absolute numbers)

**Fig. 2:**
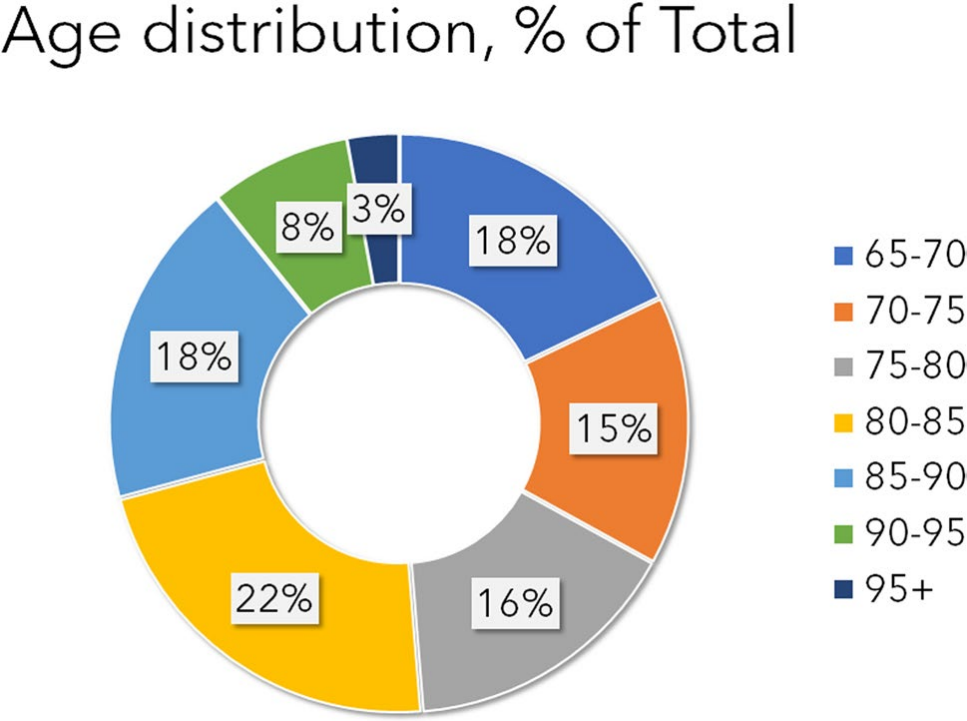
Distribution of different age groups in % of total patients (male and female together)

The average age in males (*N* = 426 patients) was 77.7 ± 8.2 y.o., of females (*N* = 547 patients) 81.1 ± 8.2 y.o. and in total (*N* = 973 patients) 79.6 ± 8.3 y.o. As shown in Figs. 1 and 2, most male patients are injured in the age group of 65–70 years (*N* = 103) and the second most in the group 80–85 years (*N* = 80). In females the age group of 80–85 years (*N* = 135) and 85–90 years are the most exposed. The most patients are in the age groups 80–85 years and 85–90 years followed by the youngest age group 65–70 years (Fig. 2; Table 1).

**Table 1:** Presenting the distribution of the patients in both genders in the different geriatric age groups. The clinically significant difference is marked with an * at the higher value (χ^2^-test, *p* < 0.05)

	Male	% Male	Female	% Female	Total	% of Total
65–70	103*	10,6%	71	7,3%	174	18%
70–75	77	7,9%	70	7,2%	147	15%
75–80	67	6,9%	86*	8,8%	153	16%
80–85	80	8,2%	135*	13,9%	215	22%
85–90	69	7,1%	110*	11,3%	179	18%
90–95	24	2,5%	53*	5,4%	77	8%
95+	6	0,6%	22*	2,3%	28	3%
Total	426		547*		973	

**Fig. 3:**
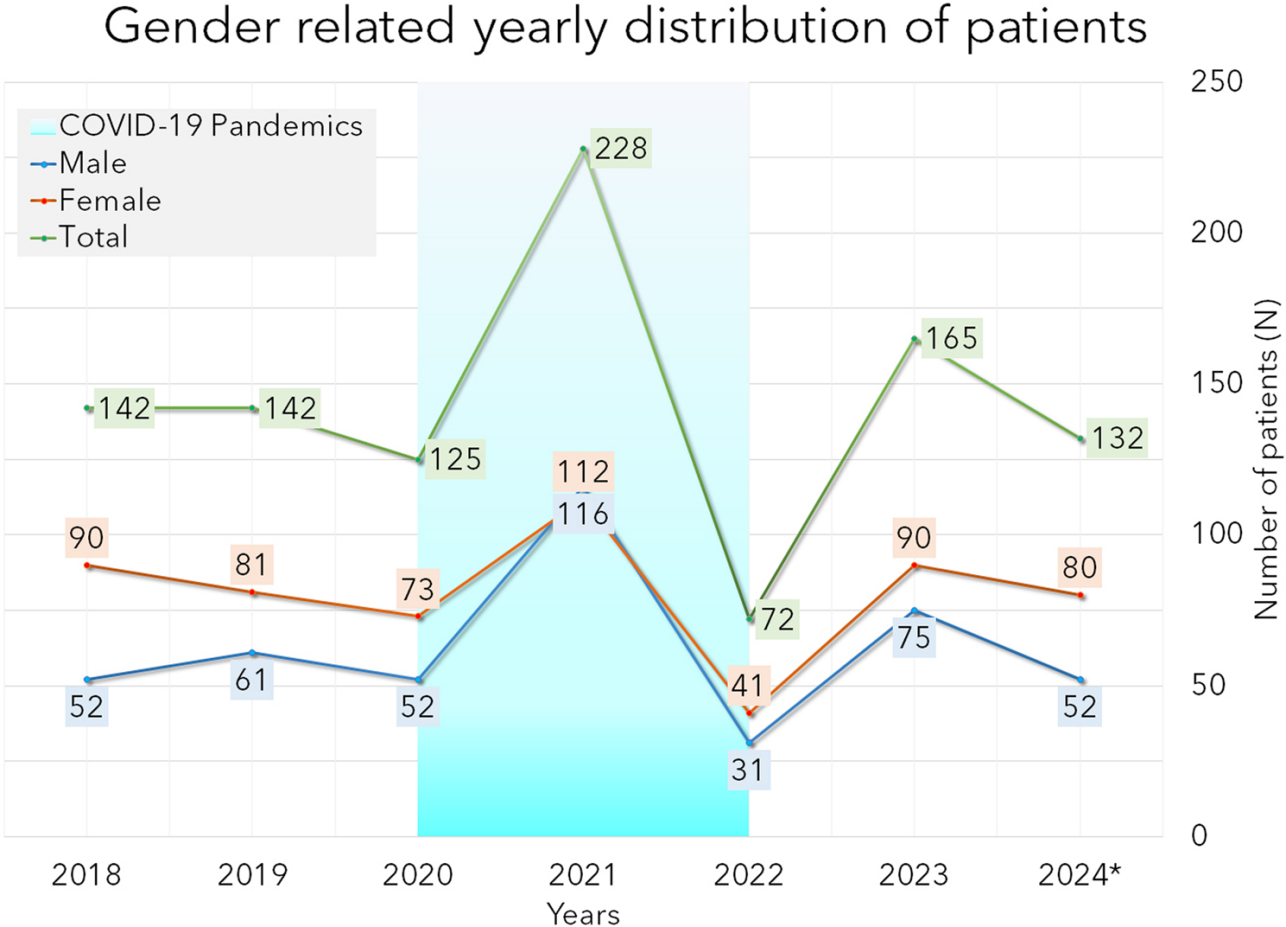
Diagram showing the yearly number of patients (2024 extrapolated, see text), blue highlight marks the pandemic years

Figure 3 shows the number of cases per year in the three periods. In females, a 30% increase and in males, a 73% increase were observed in 2021. In the last year of the pandemic (2022), a record-breaking decrease in the case numbers could be recognised. It is also remarkable that in 2021, the trend was reversed: more males than females were affected. In all other years, significantly more females than males were seen (χ^2^-test, α = 0.05). The comparison of the data from the complete database and geriatric population using the Wilcoxon ranked test showed a significant similarity (*p* = 0.009).

Table 2 summarizes the etiology of all head and neck injuries. The most cases (804 of 973, 82.6%) were falls. Road traffic accidents (72 of 973 cases, 7.4%) represented a significant part. Other cases, including work-related accidents, sports trauma, or interpersonal violence, were rare. Interpersonal violence was seldom the cause before and after the pandemic (0 or 1 case yearly), but in 2021–2022, respectively, 5 and 4 cases were reported.

**Table 2:** Summarizes the ethiology of the injuries

Ethiology	*N*
Fall	804
Road traffic accident	72
Other	58
Interperonal violence	12
Work accident	10
Sport accident	6
Unknown/Missing	11

The concomitant diseases (Table 3) played an essential role in this population: 622 of the 973 patients had hypertension (64%), 368 (38%) had diabetes or other endocrine diseases, 211 had ischemic heart disease (22%) and 51 (5%) had a myocardial infarction. Additionally, 174 neurological diseases (18%) and 152 (16%) cases of dementia. Further 901 concomitant diseases were recorded. Thus, a total of 2479 concomitant diagnoses correspond to 2.6 per patient. 514 of the 973 patients required anticoagulant therapy: 213 took aspirin, 35 vitamin-K antagonists, and 216 novel oral anticoagulants (NOACs).

**Table 3:** Representing the concomittant diseases. Please note that the end result of the table is higher than the total number of patients, as one patient May present with more than one concomittant diseases. The table presents the different conditions by frequency

Hypertension	622
Ischemic heart disease	211
Endocrin	196
Neurological disease	174
Diabetes, endocrin	172
Dementia	152
Oncological disease	75
Eye disease	71
Valvular heart disease	61
Abdominal diseases	55
Acute myocardial infarction	51
Hematological diseases	23
Acute bleeding, not otherwise classified	17
Other	599
Total	2479

**Fig. 4:**
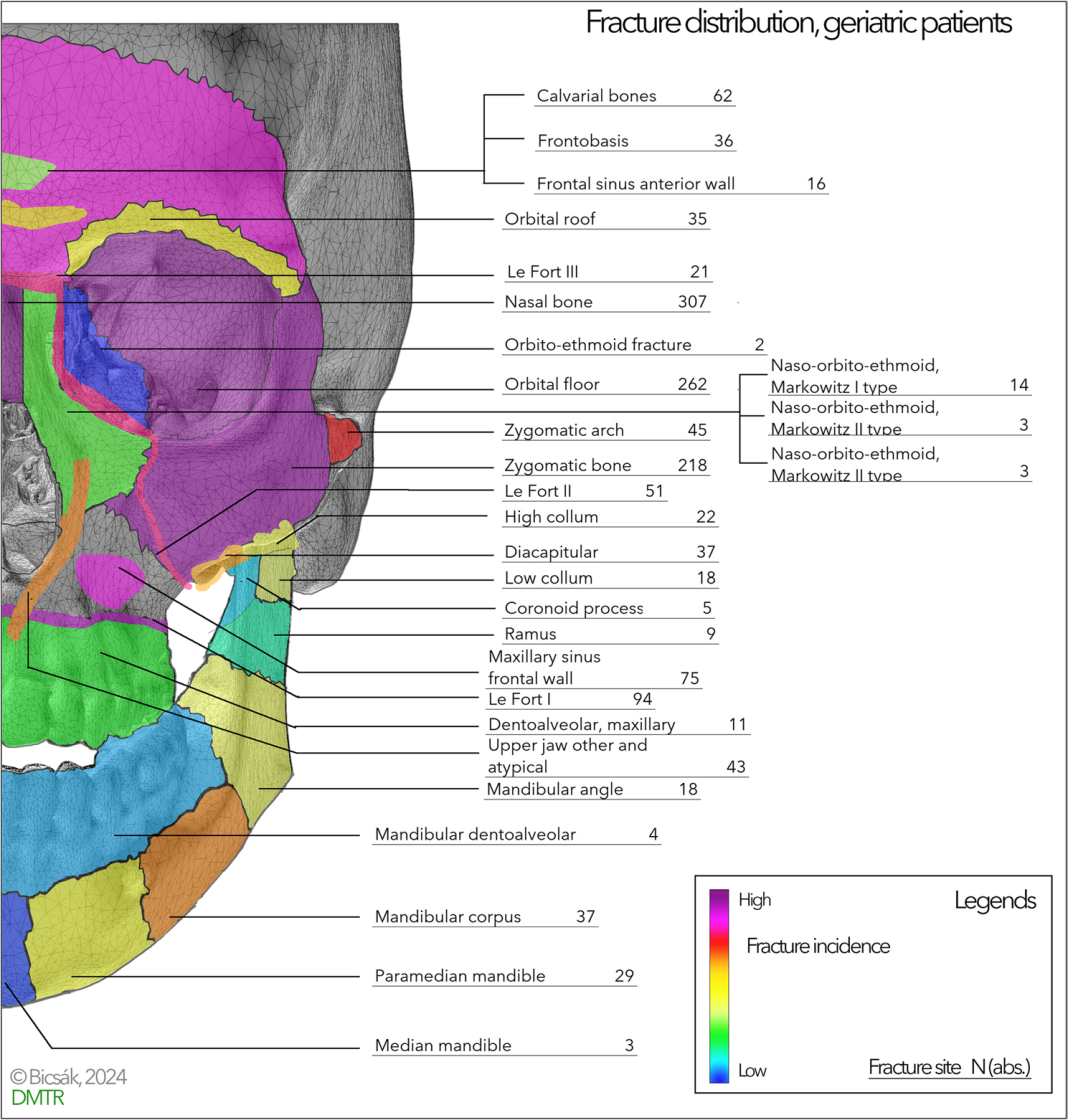
Summary heat-map of the fracture site distribution in elderly. Please, note the dominance of the forehead and midface fractures

Table 4 presents the summary of the different injuries in geriatric head and neck trauma. 480 (49.3%) patients had a cerebral injury, 360 (36.9%) sustained significant soft tissue injuries that required in-hospital stay or occurred as concomitant injuries to the head and neck region. 98 isolated fractures were found in the upper face region, 710 in the midface region. 125 isolated fractures of the mandible, and 44 panfacial fractures were reported. To treat those injuries, 509 operations under general anaesthesia were performed (52%). A detailed fracture classification is presented in Fig. 4. It is remarkable in the image that – besides the nose – the lateral midface, forehead and the frontal wall of the maxillary sinus were the most vulnerable areas. In contrast, the dentoalveolar regions of the jaws, the median mandible were rarely affected.

**Table 4:** Presents the list of injuries listed by frequency. One patient May present with more than one injury

Head and neck injury type	*N*
Midface	710
Cerebral injury	429
Soft tissue injury	360
Lower face	125
Upper face	98
Panfacial fractures	44

## Discussion

The Dortmund Maxillofacial Trauma Registry provides up-to-date results on geriatric head and neck trauma. Our main findings can be summarized in three points: (1) the demographic situation of geriatric head and neck injured patients, (2) changes during and after the SARS-CoV-2 pandemic, and (3) fracture distribution.

### Demographics

Generally, geriatric patients with head and neck injuries can be divided into two major age groups. The younger age group from 65 to 75 years old shows similar gender distribution to the distribution of younger age groups, with male predominance [13]. The difference is clinically significant in the age group of 65–70 years (χ^2^-test, *p* < 0.001). In the age group of 70–75 years the difference is yet marked but not clinically significant (*p* = 0.414). Above the age of 75 years the trend is inverted and more females are injured than males. Female predominance is clinically significant in all subgroups.

The demographic situation was approximately stable before the pandemic. As seen in Fig. 3, the yearly number of injuries started to show very high volatility from the beginning of the pandemic. This has led to unpredictability in patient care planning and sometimes to partial overload, sometimes to low usage of beds. However, as seen, 49.3% had concomitant cerebral injury. This necessitates inpatient observation with further diagnostics. Also, concomitant diseases must be treated: 2.6 per capita have been registered in very severe manifestations in some cases.

Our data are highly similar to those in the literature. Kannari et al. reported 82% falls [14]. In another study from Finland, a similar trend was observed over 15 years [15]. In other societies with fewer elder patients (5% among head and neck injured) and more in working age, the aetiology shifts to high falls, work, and traffic-related accidents [16–18]. In the United States, literature data indicate that 25% of the elderly suffer from falls [19]. Aetiology correlates with age [20]. However, in this dataset, 46% were above 80 years old, while in our study, 51%. The rate of surgical treatment ranges from 5% to 26.7% (Zelken et al., 2014; Kannari et al., 2022; Shumate et al., 2017); in our study it was 52%. Multimorbidity of elderly patients is an important issue. A Swiss study showed that 73.9% of the patients received antiplatelet therapy and 26.1% oral anticoagulants [21]. Despite the slightly different rates, both literature data in our study highlight this important point. In the Swiss study, a higher number of vitamin K antagonists were used; in the German setup, more people received NOACs. The resulting hematomas may alter the diagnosis by covering other fracture signs. Winstead et al. found cardiovascular disease as the most common cause of falls, followed by endocrine disease, neurologic disorders, and musculoskeletal problems [19]. In our study, similar results were shown (hypertension, ischemic heart disease, infarction etc., preceding endocrine and diabetes, and neurologic disorders).

### Time course, changes of the patient numbers

The first year (2020) of the pandemic led to a drop in the case numbers in both genders. The second year (2021) led to an extreme high in females, + 30%, and in males, + 90%, and the third year (2022) led to record-low case numbers. In the post-pandemic period, this high volatility seems to remain present, making the needs less predictable.

It is a question of why this volatility was observed. Lockdowns, complete close-down of elective care, the need to provide a negative test to enter healthcare facilities, and fear of getting sick may explain the countrywide dip in the case numbers [22]. Unfortunately, in this paper by Meisgeier et al. data is available until 2021. The nationwide dip in the case numbers during the first period of the pandemic is similar to our observation: 24.2% decrease in the upper face region, 14.3% decrease in the midface, and 6.1% decrease in the mandible (all age groups) [22]. The extremely high peak in the following year might be a rebound effect of missing or decreased elective capacities that reduce the level of primary care, ending in an escalation of chronic conditions. Social isolation may also play an important role: relatives were advised to keep their distance to avoid infecting older family members. This automatically means less support and less perception of decreasing general condition.

What is interesting is the much higher increase in males (90% vs. 30%, respectively). Most literature states older women as a very vulnerable group, yet our data suggests an easy escalation of the situation in men under modified circumstances.

Social stress, fear, and anger can be seen due to the increase in interpersonal violence cases (despite social distancing). A review by van Houten et al. found that most literature data suggests the head and neck region as the primary predilection of elder abuse [23].

### Fracture distribution

Predominant fracture sites are the nose, the lateral midface (orbital floor (262), the zygomatic bone (218), the LeFort I level (94), fractures near the cranial base (108 fractures in total), and the isolated fractures of the anterior wall of the maxillary sinus (75)). The predominance of falls in the etiology explains the high frequency of zygomatic bone, orbital floor, and nasal bone fractures and the high frequency of cerebral trauma. The fractures of the mandible are much rare and match the biomechanical expectations. Compared with our prior study data and literature, the actual fracture distribution fits the trends. The fracture distribution with dominant midface corresponds to literature data [21, 24–28].

There is a trend to underdiagnose the elderly [14], especially if they live in nursing homes or are hospitalized. We found the rate of cervical spine injuries increases with age; female gender is a predisposing factor [29]. Therefore, a thorough initial body check and a CT scan of the head and cervical spine are very important. Similar reports have recently emerged from many countries of the world [27, 30–32]. Shumate et al. found 54% cerebral injuries and 9% spinal injuries; among them, 75% were found in the cervical spine (Shumate et al., 2017), Zelken et al. reported 5.3% cervical spine injuries, and 20.9% other spine injuries, 63% brain injuries [28].

Our study has shown a significant feature of geriatric head and neck trauma: the volatility of case numbers has been extreme since the pandemic. Thus, healthcare providers must check their systems to be able to react to intermittent increases in elderly case numbers. In nearly half of the geriatric population, anticoagulant therapy can be expected. Also, an average of 2.6 concomitant diseases per patient is present; these are, in many cases, severe and come in combinations. Providing on-time diagnosis and sufficient combined treatment for head and neck injuries and associated medical conditions poses an exceptionally high challenge, even in highly specialized trauma centres.

### Limitations of the study

One drawback of the study is its single-centre nature. Even with a high patient number represents one single region. To compensate for this, the comparison of our data with the database of German Association of Traumatologists (DGU TraumaRegister ^®^) has shown that our data for the general population is a valid probe for the populations of Germany, Austria and Switzerland [33]. Extrapolating this data and taking the above mentioned similarities in consideration, we can assume a good representation of the Central-European population data and trends in an urbanized area.

The definition of geriatric age/elderly is not unified in the literature. Some define geriatric age as 60 years and older [34], others as 70 years and older. Both the OECD [35] and German consensus paper [11] acknowledge 65 years and older.

This is one of the first studies providing data from the pre-pandemic, pandemic and post-pandemic periods. The comparison with other centres or studies is often just partially possible.

## Conclusion

In summary, the following points highlight our study:

- the volatility of case numbers have been extreme since the SARS-CoV-2 pandemics. Males were much more affected than females (in contradiction to literature).
- elderly with head and neck injuries require special attention in appropriate trauma centres.
- the fracture distribution tends to highly exposed regions, like the nasal bone, zygomatic bone and orbital floor.
- There is a high rate of cerebral injuries and anticoagulant therapy.
- half of the patient requires operative treatment for their fractures.
- due to predictable socioeconomic changes, the geriatric population remains a vulnerable one.

Further trends in the forthcoming years should be closely monitored. If other specialties describe similar tendencies, acute measures will be inevitable, as problems with vulnerable populations may indicate deeper problems.

## Data Availability

Data availability is governed by the Data Protection laws of the European Union, the Federal Republic of Germany, the State of North-Rhine-Westphalia, and the regulations of Dortmund General Hospital. Data is, therefore, purposely made available on-site.

## Abbreviations

AO: Allgemeine Osteosynthesegruppe
CT: Computed tomography
NOACs: Novel oral anticoagulants
OECD: Organisation for Economic Co-operation and Development
